# Induction of the GABA Cell Phenotype: An In Vitro Model for Studying Neurodevelopmental Disorders

**DOI:** 10.1371/journal.pone.0033352

**Published:** 2012-03-21

**Authors:** Sivan Subburaju, Francine M. Benes

**Affiliations:** 1 Program in Structural and Molecular Neuroscience, McLean Hospital, Belmont, Massachusetts, United States of America; 2 Department of Psychiatry, Harvard Medical School, Boston, Massachusetts, United States of America; 3 Program in Neuroscience, Harvard Medical School, Boston, Massachusetts, United States of America; Hospital Clinic - University of Barcelona, Spain

## Abstract

Recent studies of the hippocampus have suggested that a network of genes is associated with the regulation of the GAD_67_ (GAD1) expression and may play a role in γ-amino butyric acid (GABA) dysfunction in schizophrenia (SZ) and bipolar disorder (BD). To obtain a more detailed understanding of how GAD_67_ regulation may result in GABAergic dysfunction, we have developed an in vitro model in which GABA cells are differentiated from the hippocampal precursor cell line, HiB5. Growth factors, such as PDGF, and BDNF, regulate the GABA phenotype by inducing the expression of GAD_67_ and stimulating the growth of cellular processes, many with growth cones that form appositions with the cell bodies and processes of other GAD_67_-positive cells. These changes are associated with increased expression of acetylated tubulin, microtubule-associated protein 2 (MAP2) and the post-synaptic density protein 95 (PSD95). The addition of BDNF, together with PDGF, increases the levels of mRNA and protein for GAD_67_, as well as the high affinity GABA uptake protein, GAT1. These changes are associated with increased concentrations of GABA in the cytoplasm of “differentiated” HiB5 neurons. In the presence of Ca^2+^ and K^+^, newly synthesized GABA is released extracellularly. When the HiB5 cells appear to be fully differentiated, they also express GAD_65_, parvalbumin and calbindin, and GluR subtypes as well as HDAC1, DAXX, PAX5, Runx2, associated with GAD_67_ regulation. Overall, these results suggest that the HiB5 cells can differentiate into functionally mature GABA neurons in the presence of gene products that are associated with GAD_67_ regulation in the adult hippocampus.

## Introduction

Decreased expression of the 67 kDalton isoform of glutamic acid decarboxylase (GAD_67_) in the hippocampus has been reported in numerous studies on schizophrenia and bipolar disorder [1], [2], [3], [4], two disorders that are thought to be neurodevelopmental in nature. A complex network of genes plays a role in the regulation of GAD_67_ and shows uniquely different expression patterns in the two disorders [3], [5], [6]. To understand how the differentiation of GABAergic cells may contribute to dysfunction in neurodevelopmental disorders, it is imperative that novel methods are developed for studying how the GABA cell phenotype is generated and maintained during the pre- and postnatal periods in distinct brain regions. GABA cells in the hippocampus develop in response to a finely tuned temporal and spatial pattern of signals emanating from the surrounding cells/tissues at different stages of phenotypic differentiation [7]. These clues are fundamentally different between cells developing in different regions, such as the hippocampus or striatum, and the same stimulus can provoke different phenotypic outcomes in GABA cells of different regions, depending upon the collective effect of stimuli acting on them at a given point in their life cycle [8], [9], [10]. Thus, in using a cell culture model to study GABA neurons, it is imperative that these cells are phenotypically similar to those endogenously present in the region under study. Toward this end, we have established a novel in vitro model in which multipotent progenitor cells in HiB5 hippocampal cultures are differentiated into mature neurons that express GAD_67_ and other genes associated with the GABA phenotype in the adult hippocampus.

An important strength of the in vitro model described below is that proliferating progenitor cells allow for the production of large numbers of cells that can be driven towards a specific neuronal phenotype, like that of GABAergic interneurons. Central nervous system progenitor cells can give rise to both glia and neurons. Conditionally immortalized progenitor cells have a consistent lineage that allows them to differentiate into a cell line with a specific phenotype. A number of GABAergic cell lines of striatal or mesencephalic origin that express GAD_67_ and produce GABA in vitro have been generated and used for molecular studies of GABA cell regulation [11], [12], [13], [14], [15]. However, a GABAergic cell line derived from the hippocampus has not as yet been developed and will be of critical importance for in vitro modeling of GABA cell differentiation and functional regulation, as it relates to this region. Since the cellular environment, which influences the development of a cellular phenotype, is different in distinct brain regions, it can be safely be assumed that hippocampal GABA neurons are different from those of striatal or mesenchymal origin. The HiB5 progenitor cell line has been derived from rat hippocampus taken at embryonic day 16 (E16) [16] using the temperature-sensitive tsA58 allele of the SV 40 large T antigen.

This HiB5 cell line has presented itself as a promising starting point to produce hippocampal GABAergic neurons in vitro, so that genes involved in the regulation of GAD_67_ can be studied under highly controlled conditions. This model can be used to characterize cellular and molecular changes associated with the maturation of GABA cells and eventually develop cell-based therapies [17], [18] for disorders in which GABA cell dysfunction plays a central role.

## Materials and Methods

### Cell culture, differentiation protocols and mRNA evaluation

Neural precursor SV40 large T antigen-immortalized HiB5 cells originally prepared from Sprague Dawley rat embryonic (E16) hippocampus [16] were kindly provided by Prof. Anders Björklund, Lund, Sweden. The cultures were maintained in proliferation medium consisting of Dulbecco's Modified Eagles Medium (Invitrogen) supplemented with 10% fetal calf serum (FCS; Gibco-BRL) in 1% Penicillin-Streptomycin and a 5% CO_2_ environment at (33°C). To induce and maintain differentiation, cells were incubated at 39°C in N2-supplemented serum-free medium (DMEM/F12 1∶1) plus additional neutirents. This change in conditions was generally accompanied by a low amount of cell death.

**Figure 1:**
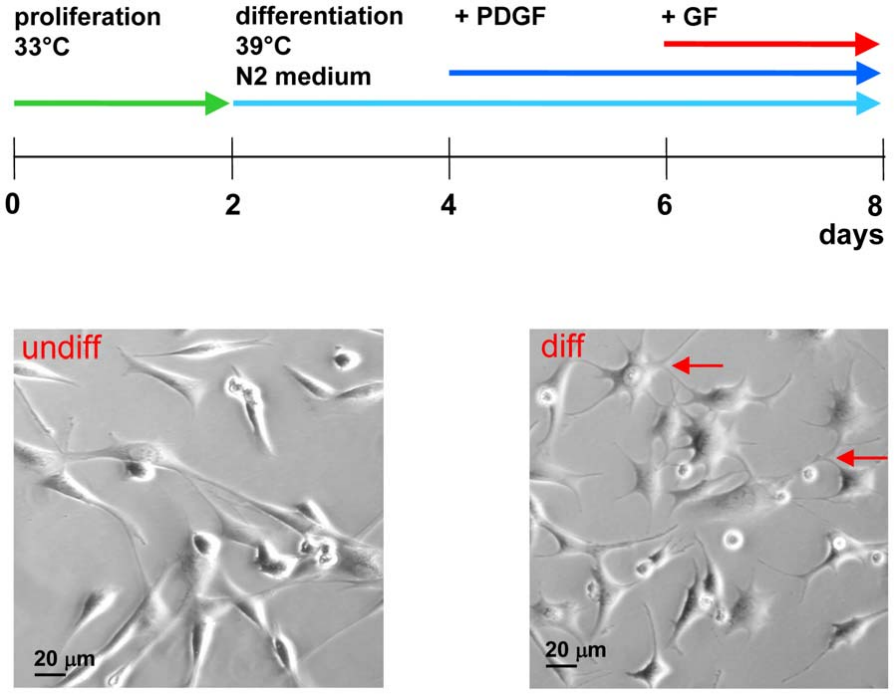
Treatment scheme for chronic influence of growth factors on expression of GABA cell marker. Cells were maintained for two days under proliferation conditions (undifferentiated (undiff) as shown in the left picture). For the first 4 days of differentiation, a routine differentiation protocol for HiB5 was followed (see methods section): after two days at 39°C in N2 medium, PDGF (30 ng/ml) was added and the cells incubated for another 2 days. To study the influence of growth factors they were added at day 4 and cells incubated for another 2 days.

Various differentiation factors were tested for their potency to induce GAD_67_ expression For basal culturing, an established protocol for inducing differentiation of HiB5 cells towards a neuronal phenotype was used [19]. After 2 days at 39°C, platelet derived growth factor (PDGF; 30 ng/ml; Sigma) was added and the cells incubated for another 2 days. To evaluate the influence of treatment with growth factors on differentiating HiB5 precursor cells, PDGF (30 ng/ml), 50 or 100 ng/ml BDNF, 50 ng/ml NRG-1, or 10 ng/ml VEGF was added to the media containing 30 ng/ml PDGF for an additional 2 days. For the details of the treatment scheme, refer to Figure 1. Total RNA was extracted and subjected to quantitative RT-PCR. The growth factor concentrations employed were based on those described in previous reports [20], [21], [22], [23], [24], [25], [26], [27]. None of the growth factors induced higher GAD_67_ mRNA expression levels than the others. Therefore, we decided to use the established protocol with PDGF stimulation in further experiments. Since BDNF had been shown before to induce morphological neuronal differentiation in HiB5 cultures and a neuronal phenotype was the second criterium for GABAergic neuron phenotype, we stimulated the cells with BDNF in addition to PDGF and analyzed GAT1 and GAD_67_ mRNA and protein expression. Although the addition of BDNF did not increase GAT1 or GAD_67_ further than PDGF alone, all further experiments were conducted in the presence of PDGF and BDNF (50 ng/ml) to ensure differentiation towards neuronal morphology.

### Phenotype characterization

Cells were analyzed for expression of GAD_67,_ and GAT1 mRNA using the TaqMan qPCR gene expression assay system. Briefly, total RNA was isolated from HiB5 cells using the Trizol reagent (Life Technologies). RNA was purified using RNeasy kit reagents (Qiagen) and subjected to RNase-free DNase digestion. The concentration and quality of total RNA was determined spectrophotometrically. cDNA was generated from 1–2 µg RNA per sample using the Superscript III Vilo cDNA synthesis kit (Invitrogen) or High capacity cDNA reverse transcription kit (Applied Biosytems). The expression levels of mRNA for various genes were measured by real-time PCR using TaqMan Gene Expression assays on Chromo4 Continuous Fluorescence Detection System (BioRad). For each sample and gene, three replicates were run in a 96-well plate. The probes contain a 6-carboxy-fluorescein phosphoramidite (FAM dye) label at the 5′ end of the gene and a minor groove binder and nonfluorescent quencher at the 3′ end and are designed to hybridize across exon junctions. β-2-microglobulin (VIC dye) was used as an endogenous control. Real-time PCR reactions were carried out using 1–2 µl of cDNA for each reaction in a 20 µl volume following the manufacturer's protocol. Gene expression values were determined as Δ*C*_t_ (*C*_t_−*C*_t 18s_) and fold changes between different samples were determined as ΔΔ*C*_t_ (Δ*C*_t vehicle_−Δ*C*_t_)_._

### Immunocytochemistry

The differentiated cells were fixed with ice-cold 4% paraformaldehyde in 0.1 M phosphate buffer (pH 7.4) for 20–30 minutes and a standard immunofluroescence technique was used to visualize various neuronal and molecular markers. Antibodies used included monoclonal: anti-b-III-tubulin 1∶1000 (Sigma); anti-GAD_67_ 1∶1000 (Sigma); anti-PSD95 1∶400 (Sigma); anti-Pax5 1∶40 (BD Pharmingen); anti-Runx2 1∶200 (R&D Systems); antiGAD65 1∶50 (Sigma); anti-parvalbumin 1∶100 (Sigma); and polyclonal: rabbit anti-GABA 1∶2000 (Sigma); rabbit anti-GAT1 1∶100 (Phosphosolutions); rabbit anti-HDAC1 1∶200 (Upstate Biotechnology); rabbit anti-DAXX 1∶200 (Santa Cruz); rabbit anti-MAP2 1∶500 (Millipore); anti-calbindin 1∶50 (Millipore); rabbit antiGluR5 1∶50 (Millipore); rabbit antiGluR6/7 1∶50 (Millipore). Fluorescence (Alexa)-coupled secondary antibodies were raised in donkey (Invitrogen) and used at 1∶600. A Leica fluorescent or Leica TCS/SP2 confocal microscope was used to evaluate fluorescent staining using appropriate excitation beams and emission filters. For confocal studies, all analyses were performed using the sequential scanning mode to avoid channel bleeding and the detection of spurious double labeling.

### Cell Counting

All cell counts were performed on codified samples under strictly blind conditions. In cell culture, 100 DAPI-positive nuclei were expressed as the total number of cells in each condition. Total cell number and the percent total counts for GABA-, acetylated-tubulin- or nestin-positive neurons were determined using cells grown on individual coverslips representing each marker and were expressed as the number of cells per marker for undifferentiated versus differentiated cells. The cell numbers were expressed as means ± standard deviations (SDs) for 5 independent experiments. The statistical differences between the groups were determined using a one- way ANOVA.

### Western Blot

Cultured cells were washed with ice-cold PBS and harvested in lysis buffer (T-Per tissue protein extraction reagent, Pierce) containing 10 µl/ml protease inhibitor cocktail and 10 µl/ml phosphatase inhibitor cocktail I and II each (Sigma-Aldrich). Protein extracts were stored at 80°C. Fifty micrograms of protein extracts from undifferentiated or differentiated HiB5 were separated on 10% SDS-polyacrylamide gels and transferred onto a PVDF membrane (BioRad). Protein expression of markers for the GABAergic phenotype and other proteins of interest were assessed using specific antibodies and standard Western blot procedures. The antibodies employed were specific for GAD_67_ (1∶1000; Sigma), GAT1 (1∶100; Phosphosolutions), or β-actin (1∶5000) overnight at 4°C. Bands were visualized using chemiluminescence (ECL Western Blotting Analysis System and ECL Hyperfilm, Amersham Biosciences). For statistical evaluation, blots were scanned and analyzed using the public domain NIH image program. Data were normalized using the respective loading controls.

### High-Performance Liquid Chromatography Analysis of In Vitro GABA Synthesis

To examine the GABA release by HiB5 cells, cultured cells were differentiated in (100×20 mm^2^) dishes as described above. GABA release (into the medium) was examined by high-performance liquid chromatography (HPLC) and detection with a Coulochem III electrochemical detector to determine the basal or stimulated level of GABA release into the medium.

After three rinses with artificial cerebrospinal fluid (aCSF) (containing in mM: NaCl, 124; KCl, 3.0: KH2PO4, 1.25; CaCl2, 2.0; MgSO4, 1.0; NaHCO3, 26; glucose, 10; pH 7.4), the cells were incubated with aCSF for 3 min. Supernatants were collected and referred to as basal (time 0). Next, cells were depolarized for 15 minutes with 100 mM KCl (dissolved in aCSF) at 37°C. The supernatants were collected and the concentration of GABA in the medium determined by HPLC. Measurement of GABA was performed by ESA Biosciences Inc. (Chelmsfords, MA, USA). Briefly, derivatization with o-phthalaldehyde and 2- mercaptaethanol was followed by reverse-phase isocratic liquid chromatography with electrochemical detection.

## Results

### Evaluation of the influence of various growth factors on GAD_67_ expression in HiB5 cells

**Figure 2:**
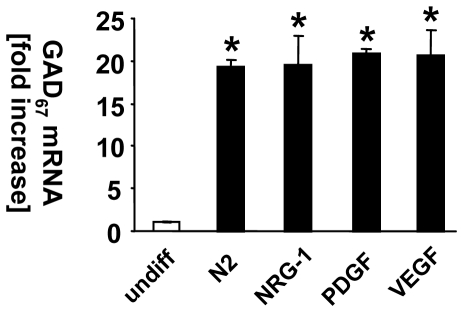
Screening of growth factors for GAD_67_ increasing activity in differentiated HiB5 cells. Cultured HiB5 cells were switched to differentiation conditions. In the presence of PDGF, the cultured cells were treated with NRG-1 or VEGF and cells were incubated for 2 additional days. Controls were cultured in N2 medium with PDGF alone. Cells were harvested in Trizol, the total RNA extracted and subjected to quantitative RT-PCR. Data are expressed as fold change of N2 control. (*≤0.001 vs undiff).

Cell fate is determined by growth factor types, concentrations and respective timing of treatment. To evaluate which growth factors drive HiB5 cell differentiation towards a GABA phenotype when applied acutely, cells were kept under differentiation conditions (39°C, N2 medium) for 2 days, incubated for 2 more days in the presence of PDGF to drive differentiation towards a neuronal fate and then stimulated with different growth factors (BDNF, NRG-1 or VEGF) two more days in the presence of PDGF (treatment scheme Figure 1). Expression of the GABA synthesizing enzyme GAD_67_ was measured using quantitative RT-PCR. Differentiation conditions alone without acute growth factor addition induced GAD_67_ mRNA expression significantly. Neither NRG-1 nor VEGF or additional PDGF further increased the expression of GAD67 mRNA above the level of cells cultured in differentiation medium alone (Figure 2). Results for BDNF are shown below.

### Expression of Markers for Neuronal Maturation in Differentiated HiB5 cells

**Figure 3:**
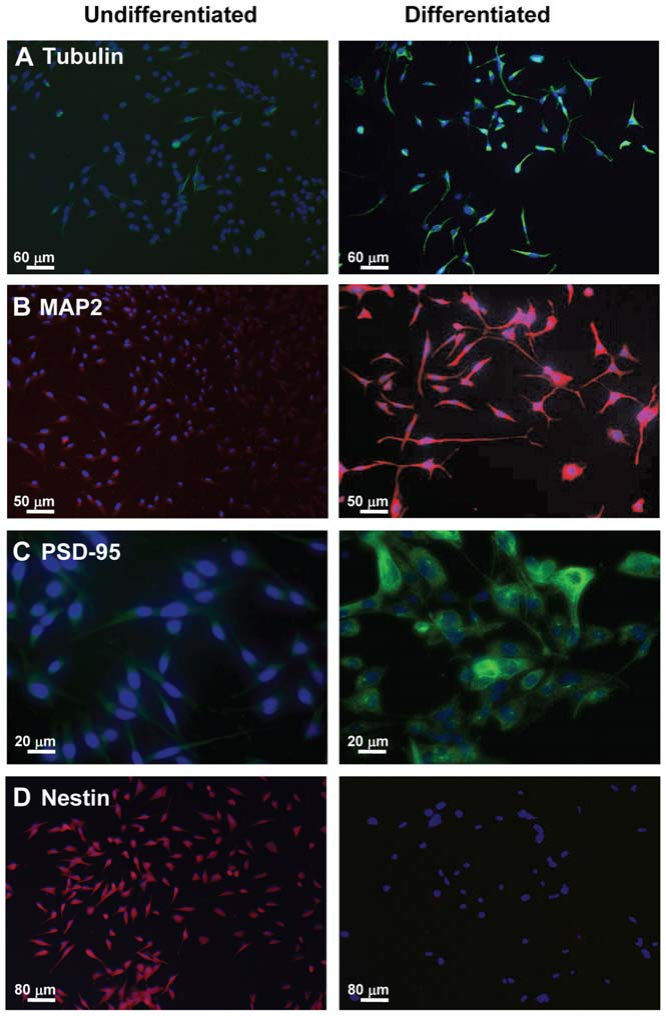
Expression pattern of mature neuron markers acetylated tubulin, MAP2 and PSD-95 and stem cell marker nestin in undifferentiated and differentiated HiB5 cells. Specific markers were detected using primary antibodies and immunofluorescence detection with DAPI as nuclear counter stain. Detectable levels of IF were associated with, acetylated tubulin (**A**); MAP2 (**B**); PSD-95 in differentiated HiB5 cells (**C**), while nestin (**D**) is only seen in undifferentiated cells.

To ensure that the differentiated HiB5 cells expressed a neuronal phenotype, mature neuron markers acetylated tubulin, MAP2 and post-synaptic density protein 95 (PSD-95) were employed in an immunofluorescence (IF) analysis of differentiated vs. undifferentiated neurons. Additionally, the stem cell marker nestin was used as control. Differentiated HiB5 cells showed increased amounts of acetylated tubulin, MAP2 and PSD-95 compared with undifferentiated cells (Figure 3 A–C), showing that differentiation conditions resulted in mature neurons. Undifferentiated cells, in contrast, showed high levels of nestin expression, which was virtually undetectable in differentiated cells (Figure 3D). Quantitative evaluation of acetylated tubulin- and nestin-positive cells showed almost 100% differentiation (Table 1).

**Table 1:** Quantification of cells expressing differentiation stage-specific markers.

Marker	Undifferentiated	Differentiated
GABA	1±1	92±3*
Acetylated tubulin	2±1	94±4*
Nestin	97±3	1±1*

**The data represent 100 cells/coverslips that were analyzed using cells grown on 5 different coverslips per marker and differentiation state. Statistical significance was calculated by one-way ANOVA, followed by Tukey test.**

* : **p<0.001 for GABA undiff. vs GABA diff., tubulin undiff. vs. tubulin diff., nestin undiff. vs. nestin diff., respectively Data are expressed as means ± SD.**

### Expression of GABA Neuron-Specific Markers in Differentiated HiB5 Cells

**Figure 4:**
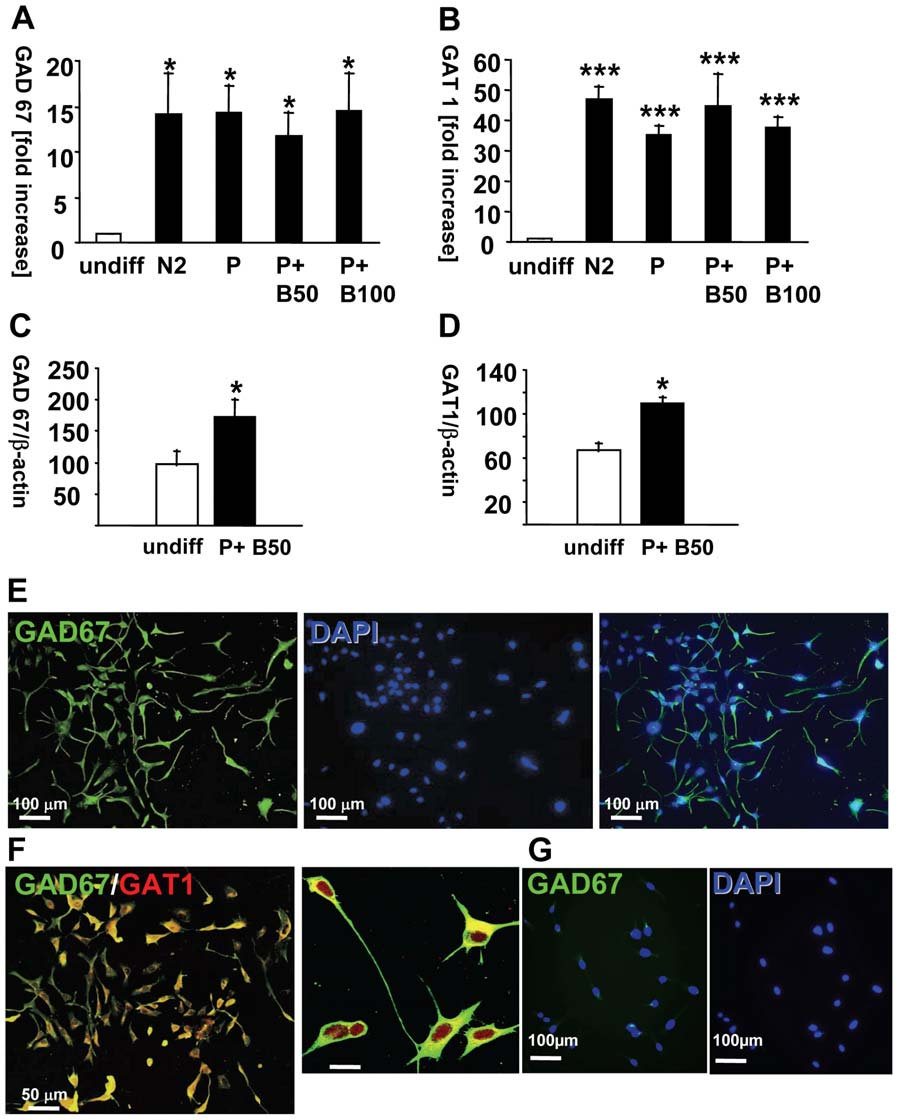
Induction of expression of key GABA cell markers GAD_67_ and GAT-1 in differentiated HiB5 cells. (**A, B**): Cells were differentiated at 39°C in N2 for two days, followed by addition of PDGF (30 ng/ml) for an additional two days. This was followed by stimulation for two days with growth factors. Quantitative (q) RT-PCR for GAD_67_ or GAT1 was used to measure changes in mRNA expression in the presence N2 supplement plus PDGF alone; additional PDGF (P); or a combination of additional PDGF and BDNF (B; 50 and 100 ng/ml respectively). Values are expressed as fold increases in differentiated cells compared with undifferentiated controls (**GAD_67_** vs undiff: *≤0.05; **GAT1**: vs undiff ***≤0.001). **C, D**: Western blot with β-actin as loading control was used for quantification of proteins. (*≤0.05 vs undiff). GAD_67_ and GAT-1 were detected using specific antibodies. (**E**): DAPI (blue) was used as nuclear counter stain for GAD_67_ containing cells (green). (**F**): GAD_67_ (green) was co-localized with GAT1 (red) in the same differentiated cells (yellow overlay color) (**G**) GAD_67_ (green) in undifferentiated HiB5 cells; DAPI (blue): cell nuclei.

To evaluate whether functional differentiation of neurons was occurring in the HiB5 cultures, HiB5 cells were treated with N2 alone, PDGF alone and combinations of PDGF and 50 or 100 ng/ml BDNF, respectively. Two specific functional markers for GABA neurons, GAD_67_ and GAT1, were evaluated in differentiated vs undifferentiated cells, using quantitative RT-PCR. Differentiated HiB5 cells expressed significantly more GAD_67_ (Figure 4A) and GAT1 (Figure 4B) mRNA. However, there was no difference between the treatment groups within the differentiated HiB5.

When a combination of PDGF and 50 ng/ml BDNF were used in subsequent experiments, Western blot analysis of GAD_67_ and GAT1 in differentiated vs undifferentiated HiB5 cells showed a significant increase in both GAD_67_ and GAT1 protein (Figure 4C, D).

To evaluate whether undifferentiated cells also show increases of GAD_67_, IF with GAD_67_ -specific antibodies was co-localized with DAPI for visualization of cell nuclei. As shown in Figure 4E, differentiated cells with numerous neurites expressed high levels of GAD_67_. In contrast, undifferentiated cells showing no neuritic processes expressed little or no GAD_67_, (Figure 4G). As evaluated by confocal microscopy, differentiated HiB5 cells showed immunofluorescence for both GAD_67_ and GAT1 (Figure 4F).

### Synthesis of GABA and Stimulus-Dependent Release from Differentiated HiB5 Cells

**Figure 5:**
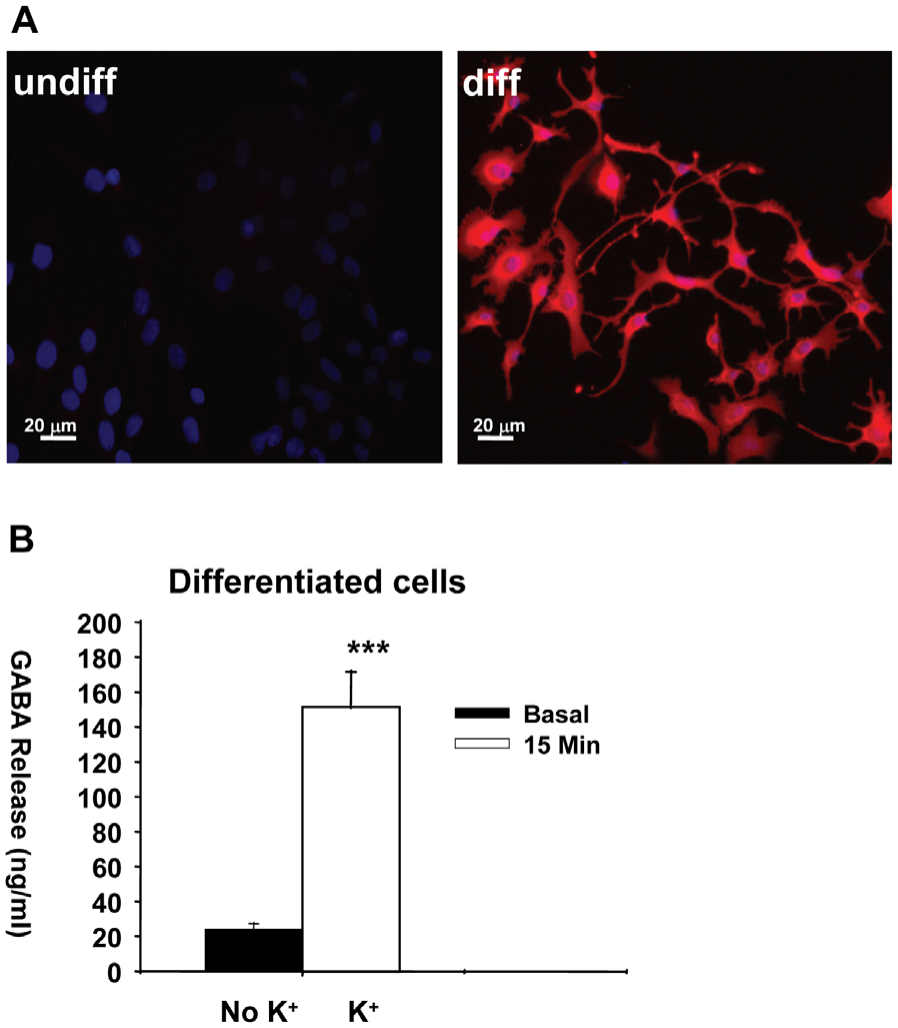
Synthesis of GABA and stimulation-induced release from differentiated HiB5 cells. (**A**) GABA was detected using a specific antibody against GABA. (**B**) GABA release was detected in artificial cerebrospinal fluid (aCSF) without KCl (basal) or with 100 mM KCl (stimulation) in the presence of Ca^2+^. GABA concentrations in aCSF were determined using HPLC. The data represent the mean ± SEM for GABA release from 6 independent culture experiments (***≤0.0004 vs baseline levels in differentiated cells).

Differentiated HiB5 cells showed high levels of GABA-immunofluorescence that was virtually absent in undifferentiated cells (Figure 5A). This seems to be in contrast with GAD_67_ expression which was detected in undifferentiated HiB5 with Western blot analysis: however, this technique is only semi quantitative and trace amounts of GAD_67_ might not be enough to produce enough GABA to be detectable with antibody staining. Both the cell soma and neuritic processes of differentiated HiB5 contained a strong GABA-IF signal. As the fibers extended with time, the intensity of GABA-IF was very intense in cell bodies. Using KCl, stimulus-dependent release of GABA from differentiated cells into the medium was evaluated, using high sensitivity chromatographic analyses of the aCSF medium. Upon stimulation with KCl (Figure 5B), differentiated HiB5 cells released a significant amount of GABA compared with basal, matching the GABA-immunofluorescence staining patterns described above.

### Further characterization of the GABA phenotype

**Figure 6:**
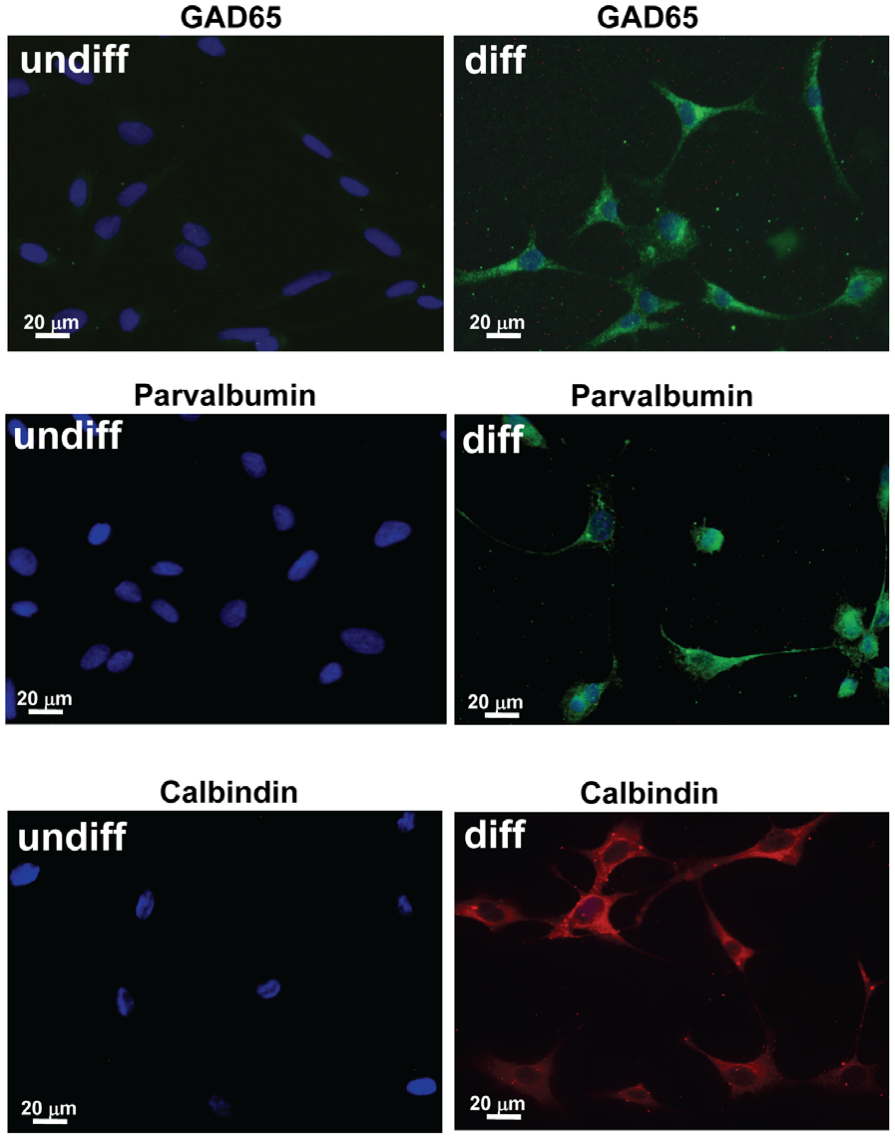
Expression of GAD_65_, parvalbumin and calbindin in undifferentiated and differentiated HiB5 cells. Antibodies were used to detect specific proteins (**A**) GAD_65_ (green) (**B**) parvalbumin (green, right) (**C**) expression of calbindin (red). Blue: cell nuclei counter stained with DAPI.

Hippocampal GABA neurons express GAD_65_, and calcium binding proteins such as calbindin and parvalbumin (PARV). To characterize differentiated HiB5 cells further, we used immunofluorescence to assess expression of GAD_65_, parvalbumin and calbindin in theses cells. All three proteins were upregulated in differentiated HiB5 (Fig. 6A–C).

### Co-localization of Genes Involved in GAD_67_ Regulatory Network

**Figure 7:**
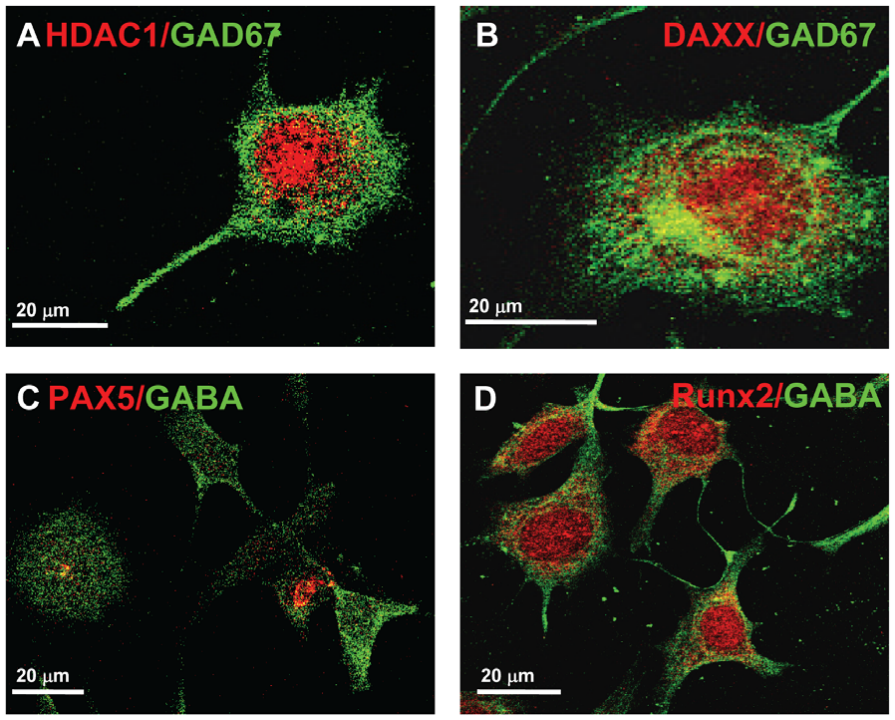
Co-localization of epigenetic regulators HDAC1 and DAXX, developmental transcription factors PAX5 and Runx2 with GAD_67_ in differentiated HiB5 cells. Specific antibodies were used to detect several proteins expressed by the target genes associated with a GAD_67_ regulatory network. GAD_67_ (green) was co-localized with (**A**) HDAC1 (red); (**B**) DAXX (red) and GABA (green) with (**C**) PAX5 and (**D**) Runx2 (red fluorescence, respectively).

**Figure 8:**
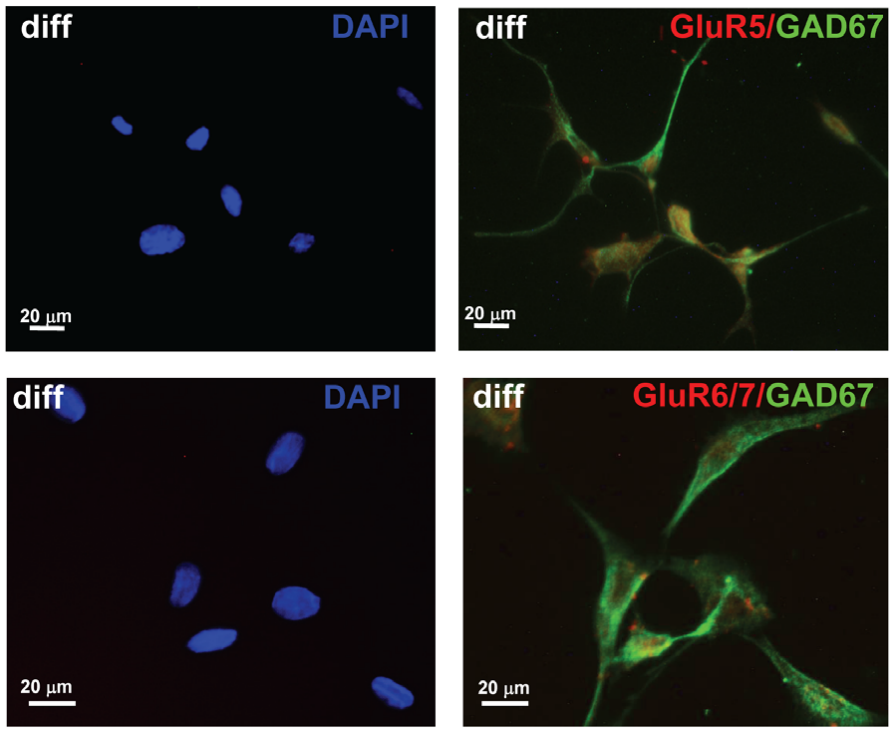
Co-localization of GAD_67_ with (A) GluR5 and (B) expression glutamate receptor subunits GluR6/7 in differentiated cells. Specific antibodies were used to detect glutamate receptor subunits. (**A**) GluR5 expression in differentiated HiB5 cells; (**B**) GluR6/7 expression in differentiated HiB5 cells. GluRs (red) was co-localized with GAD_67_ (green).

IF was used to co-localize GABA with HDAC1 and DAXX, two genes associated with epigenetic suppression of GAD_67_ promoter activity. In differentiated HiB5 cells (Figure 7 A–B), both factors were expressed in GABA-positive HiB5 cells. HDAC1-IF was localized in the nucleus of these cells, while DAXX-IF was found in the nucleus and cytoplasm. Two transcription factors, PAX5 and Runx2, also associated with a GAD_67_ regulatory network [3] were co-localized with GABA–IF in differentiated HiB5 cells (Figure 7C–D). Regulation of GAD_67_ in hippocampus in schizophrenia is also associated with the regulation of glutamate receptors. Therefore we were interested to see whether these receptors were expressed in our model system. We could detect GluR5 in differentiated HiB5 (Figure 8A). Since commercially available antibodies detected both GluR6 and GluR7, (Figure 8B), we verified by qRT-PCR that both GluR6 and GluR7 were expressed in differentiated HiB5 (data not shown).

## Discussion

The results of this study have demonstrated that a phenotype similar to that of hippocampal GABA neurons in vivo can be induced in vitro using the HiB5 cell line. This conditionally immortalized precursor cell line can serve as a basis for studying the regulation of GAD_67_ expression in hippocampal GABAergic neurons. GAD_67_ (GAD1) appears to be regulated by a complex and diverse network of genes that includes growth factors, elements of the TGFβ and Wnt signaling pathways, transcription factors and epigenetic regulation of promoters [3]. Growth factors linked to GAD_67_ regulation (i.e. BDNF, PDGF) promoted the differentiation of HiB5 towards a GABAergic neuronal phenotype.

A major advantage of using an in vitro culture system of this type is the fact that a preponderance of differentiated neurons with the GABA cell phenotype can be generated in relative isolation, making it possible to experimentally manipulate them using a variety of genetic, pharmacological and physiological strategies. Differentiated cells are a prerequisite for biochemical characterizations; however, it is difficult to obtain them in large numbers. By using immortalized precursor cells that proliferate and generate progeny, this problem can be routinely overcome [27]. Generally speaking, the differentiation into a neuronal phenotype is a combinatorial process in which intrinsic genetic programs are activated by extracellular growth factors. By controlling the type, amount and duration of exposure of specific growth factors to which precursor cells are exposed, it is possible to direct them towards a specific cell fate [28]. HiB5 are multipotent progenitor cells with the potential for developing into either glia or neurons, depending upon their extracellular environment [29], [30], [31]. As demonstrated in this report, by addition of a specific panel of growth factors that includes PDGF and, BDNF, undifferentiated HiB5 precursor cells can be pushed toward a differentiated state with a GABAergic phenotype.

The ability of HiB5 cells to develop into GABAergic neurons may be related to their having been generated from embryonic tissue. During the prenatal developmental stages E13 and E14 of a 21day gestational period in rats, undifferentiated neuroepithelial cells express the intermediate filament nestin. By E16, a wave of in vivo neurogenesis typically begins in the hippocampus and continues into the early post-natal period when post-mitotic neurons begin to appear in the ventricular and subventricular areas. By E19, the ventricular zone stops producing neurons, while the subventricular zone continues to generate both neurons and glia [31], [32], [33], [34]. HiB5 cells derived from E16 rats retain the developmental potential of their nestin-positive precursors, even when they are at the brink of differentiating into neurons. These preconditions suggest that undifferentiated neuronal precursor cells might respond optimally to extracellular cues that have the potential to drive them towards a neuronal fate. Other immortal hippocampal cell lines dissected from the rat hippocampus between E17 and E18 are capable of generating both neuronal and glial lineages [35]. Due to their origin from a later embryonic stage, however, these cells might not have the unique combination of stem cell properties and early neuronal predisposition of the HiB5 cell at E16 that enables differentiation towards a GABAergic phenotype. Multipotent stem cells can give rise to different neuronal cell types [36], [37], [38]. The transition of a cell from a multipotent progenitor state into a mature differentiated neuron can be mapped using stage-specific markers, such as nestin, a marker for undifferentiated stem cells [39]. Once mature neurofilament constituents and synaptic proteins appear in the cells [40], [41], neuronal processes begin to sprout. Typical markers for neuronal maturation of HiB5 cells, including acetylated tubulin, MAP2 and PSD-95, were present in “differentiated” HiB5 cells, supporting the conclusion that the latter had developed a mature neuronal phenotype.

Differentiation is guided by extracellular stimuli [28], and several growth factors, such as BDNF, VEGF, NRG-1 and PDGF have been linked to the differentiation of GABAergic neurons. BDNF is capable of inducing a GABAergic phenotype in hippocampal granule cells in vivo [21], [22], [42] and increases the expression of GAD_67_ in primary hippocampal cultures from the dentate gyrus obtained from adult mice [23]. Following a cerebrovascular accident (CVA), VEGF increases cortical neuron number, the formation of new GAD_67_-positive dendrites and is capable of inducing the maturation of differentiated GABA neurons [43]. In the adult brain, neuregulin-1 (NRG-1) and its receptors ErbB2,-3,-4 are expressed in neurogenic regions like the subventricular zone and the hippocampal dentate gyrus [44], [45]. Interestingly, the signaling of neuregulin 1 via its ErbB4 receptor is altered in schizophrenic patients [46], and ErbB3 expression is decreased in the frontal cortex of these patients [47], [48]. A number of earlier studies had indicated that PDGF promotes neuronal differentiation in HiB5 cells [19], [49], [50], [51], [52], [53].

Exposure to specific growth factors, such as VEGF NRG-1 and BDNF, did not further increase GAD_67_ expression in HiB5 cells when compared to PDGF alone. Even so, BDNF stimulates the differentiation of progenitor cells in HiB5 cultures towards a mature neuronal phenotype [19] and promotes early steps in the morphological differentiation and maturation of hippocampal neurons [54], [55]. BDNF has also been associated with the formation of inhibitory synapses and increases in the number and length of axons of GABAergic neurons in E16 primary cultures [42] In the current study, the inability of the growth factors to further induce the GAD_67_ gene might be related either to the fact that the cultured cells were stimulated for a relatively short period of time (2 days), or that the GAD_67_ mRNA level in the culture was already close to a maximum 4 days in differentiation conditions (2days N2, 2days N2 plus PDGF). It is not clear why other growth factors did not further stimulate the differentiation of the GABA phenotype; however, as described before, the combination of extrinsic and intrinsic factors associated with a cell stimulation decides its fate, and a growth factor may work well in one environment, but not another, because the analogous environmental conditions are different. As discussed for BDNF, GAD1 activation in differentiated HiB5 cells might have attained a maximum level of expression, once the cells were exposed to the appropriate differentiation conditions (i.e. N2 and 39°C and PDGF stimulation).

Terminal differentiation of GABAergic neurons requires the activation and regulation of many different genes that are necessary for the synthesis, vesicular packaging and release of GABA. In the current study, expression of GAD_67_ and GAT1 marked the terminal differentiation of HiB5 neuronal precursor cells and was associated with the synthesis and release of GABA from these same cells. Further, differentiated HiB5 cells expressed GAD65, parvalbumin and calbindin. GABAergic hippocampal interneurons expressing calbindin are altered in schizophrenia [56]. Parvalbumin-positive GABA neurons are involved in neural synchrony and in the altered network oscillation in schizophrenia [57]. Thus, our findings that differentiated HiB5 cells in our model are calbindin – and parvalbumin –positive further validate our model for the study of GABA cell regulation under pathophysiological conditions.

Differentiated HiB5 cells express glutamate receptor subunits which have been found to be regulated in human hippocampus in association with schizophrenia. Additionally, two epigenetic factors (HDAC1 and DAXX) and two transcription factors (PAX5 and Runx2), were also detected in the terminally differentiated GABAergic cells. These genes are associated with the regulation of GAD_67_ in GABAergic interneurons of the adult human hippocampus [3] and their appearance in the differentiated HiB5 cells coincides with the expression of GAD_67_ protein by these cells. It is noteworthy that HDAC and DAXX show increased expression in schizophrenia, while PAX5 and Runx2 show decreased transcripts in bipolar disorder, two disorders in which parallel reductions in the expression of GAD_67_ have also been reported in a layer of the hippocampus that exclusively contains neurons of the GABAergic type [3]. The molecular endophenotypes that are associated with a common cell phenotype may be quite unique in different disorders in which GABA cell dysfunction is present.

### Conclusion

Taken together, the results of this study suggest that terminally differentiated GABA cells in HiB5 hippocampal cultures may provide a useful model for studying the molecular regulation of GAD_67_ in post-mitotic GABA cells of the hippocampus, as they may relate to various types of neurodevelopmental and neuropsychiatric disorders.
